# Structural brain changes in first episode Schizophrenia compared with Fronto-Temporal Lobar Degeneration: a meta-analysis

**DOI:** 10.1186/1471-244X-12-104

**Published:** 2012-08-07

**Authors:** Bayanne Olabi, Ian Ellison-Wright, Ed Bullmore, Stephen M Lawrie

**Affiliations:** 1 Division of Psychiatry, School of Molecular and Clinical Medicine, University of Edinburgh, Royal Edinburgh Hospital, Edinburgh, EH10 5HF, UK; 2 Avon and Wiltshire Mental Health Partnership NHS Trust, Salisbury, UK; 3 Department of Psychiatry, Behavioral & Clinical Neuroscience Institute, University of Cambridge, Cambridge, UK; 4 Cambridgeshire & Peterborough NHS Foundation Trust, Cambridge, UK

**Keywords:** Schizophrenia/pathology, FTLD/pathology, Meta-analysis, Magnetic resonance imaging/methods, Humans, Brain/pathology, Brain mapping, Imaging processing, Computer-Assisted/methods

## Abstract

**Background:**

The authors sought to compare gray matter changes in First Episode Schizophrenia (FES) compared with Fronto-Temporal Lobar Degeneration (FTLD) using meta-analytic methods applied to neuro-imaging studies.

**Methods:**

A systematic search was conducted for published, structural voxel-based morphometric MRI studies in patients with FES or FTLD. Data were combined using anatomical likelihood estimation (ALE) to determine the extent of gray matter decreases and analysed to ascertain the degree of overlap in the spatial distribution of brain changes in both diseases.

**Results:**

Data were extracted from 18 FES studies (including a total of 555 patients and 621 comparison subjects) and 20 studies of FTLD or related disorders (including a total of 311 patients and 431 comparison subjects). The similarity in spatial overlap of brain changes in the two disorders was significant (p = 0.001). Gray matter deficits common to both disorders included bilateral caudate, left insula and bilateral uncus regions.

**Conclusions:**

There is a significant overlap in the distribution of structural brain changes in First Episode Schizophrenia and Fronto-Temporal Lobar Degeneration. This may reflect overlapping aetiologies, or a common vulnerability of these regions to the distinct aetio-pathological processes in the two disorders.

## Background

Schizophrenia is a disorder characterised by positive symptoms (hallucinations and delusions), thought disorder and negative symptoms (such as apathy). Brain imaging studies have identified structural changes both early in the presentation of the illness and more extensive changes later in the course of the illness [1]. The distribution of changes has been replicated between studies [2], and may be considered a ‘structural signature’ of schizophrenia within the brain. However, an adequate explanation for this spatial distribution remains elusive. Models have been proposed involving genetic factors coding neuro-protein variants resulting in abnormal development of limbic and frontal-temporal-subcortical networks [3]. However, it remains uncertain which neuro-chemical pathways are responsible and how they cause the spatial changes.

FTLD is characterised by declines in social function, interpersonal conduct, emotional blunting, speech and language abnormalities and loss of insight [4]. It is associated with degeneration of the prefrontal and anterior temporal cortices [5]. However, the topographical distribution of structural brain changes is heterogeneous amongst different patient groups, reflecting behavioral and pathological variants. Although the most common histological feature is tauopathy [6], over half of all patients, including those with a family history of the condition, have no abnormality in the tau gene or protein [7], consistent with pathological and aetiological heterogeneity.

In this study, we investigate whether there is overlap in the distribution of brain changes in First Episode Schizophrenia (FES) and Fronto-Temporal Lobar Degeneration (FTLD). There is evidence for similarities in clinical, neuropsychological and neuroimaging findings in patients with FTLD and schizophrenia [8]. In some cases, patients with FTLD have been diagnosed with a schizophrenia-like psychotic illness years before the dementia diagnosis is made [9]. Echopraxia, echolalia, aprosody of speech, utilisation behavior, ‘negative’ symptoms, self-neglect, and bizarre, compulsive, and stereotyped behaviors are well recognised in both disorders. Executive dysfunction with relative preservation of visual perception and spatial skills [7,10] and deficits in social cognition, theory of mind, empathy and affect recognition have been identified in both disorders [11].

Frontal, temporal and hippocampal atrophy [12,13] and regionally specific reductions in the anterior corpus callosum [14,15] and anterior hippocampus [16,17] have been described in MRI studies of both FTLD and schizophrenia. Frontal hypoperfusion on single photon emission tomography or positron emission tomography constitutes one of the imaging criteria for the diagnosis of frontotemporal dementia [18], and is also one of the most robust functional imaging findings in the schizophrenia literature in patients with chronic and first-episode illness [19]. There is also recent evidence that schizophrenia and FTLD co-occur in some families, suggesting the possibility of a common vulnerability to these disorders [20]. While the pathology of schizophrenia remains uncertain, there have been considerable advances in elucidating the complex and heterogeneous pathology of FTLD [21,22]. We have chosen to examine FES rather than chronic schizophrenia because the structural changes in FES may more accurately reflect the pathological changes of the disorder, and may minimize the confounding effects of long-term medication and other aspects of chronic illness. Different antipsychotics have individual volumetric effects on brain structure [23,24] and therefore, patients with FES were used in order to reduce heterogeneity.

The aim of this review is to determine the distribution of brain changes in FTLD and FES, by employing an established meta-analytic technique (anatomical likelihood estimation, ALE) [25] that is widely used for coordinate-based meta-analyses of neuroimaging data by converting the co-ordinates of peak gray matter changes from multiple published studies into spatial probability maps. However, the accuracy and extent of these maps is dependent on the total number of peak co-ordinates available from published studies. Therefore, this study employs a new statistical approach to investigate the degree of spatial correspondence between the two disorders, taking into account the greater availability of data co-ordinates for FTLD than FES. The comparison of brain changes between an individual MRI scan and maps for different disorders may become increasingly important for early diagnosis, as currently, diagnoses of psychiatric disorders are made on the basis of clinical manifestations and associated psychosocial disturbances. There are current initiatives to encourage the classification of mental disorders for research purposes, such as the RDoC (Research Domain Criteria) approach [26]. Several MRI-based studies have attempted to distinguish between patients and healthy subjects with high accuracy (ranging from 75 to 92%) [27]. Therefore, the statistical technique described in this paper for assessing spatial overlap may have wider clinical utility in the future.

## Methods

The PRISMA (preferred reporting items for systematic reviews and meta-analyses) guidelines were followed to conduct this review [28].

### Literature search

A comprehensive keyword search of EMBASE (from 1980), PsycINFO (from 1801) and Medline (from 1950), was conducted using the following search strategy. The following Boolean phrase was used: {([Magnetic Resonance Imaging] OR [MRI]) AND ([Schizophrenia] OR [schizo*] OR [FTLD] OR [Fronto-Temporal Lobar Degeneration) AND ([Voxel] OR [VBM])}. Both free-text and expanded medical subject headings were be used. The search strategy was supplemented using a cited reference search, and by inspecting the reference lists of included articles. The search was conducted in January 2011. No time span was specified for date of publication.

### Criteria for inclusion/exclusion

Studies were considered for the review using the following inclusion criteria: 1) they were published in English as a peer-reviewed article (rather than a letter, abstract, or case report); 2) they compared a sample of formally diagnosed subjects with a group of unrelated healthy control subjects; 3) they utilized voxel-based analysis of gray matter in structural MRI scans to investigate differences in whole-brain; and 4) they reported the three-dimensional co-ordinates of changes in stereotactic space.

FTLD studies were considered if they consisted of subjects with Fronto-Temporal Lobar Degeneration or related diagnoses, such as Semantic Dementia. FES studies were considered if they included a group of subjects with schizophrenia or related diagnoses, specifically first episode schizoaffective disorder or psychosis. Only first-episode schizophrenia patients were included, therefore, papers must have documented that patients were experiencing symptoms related to psychosis without a prior diagnosis without administration of prior antipsychotic medication in order to be included in our meta-analysis.

Studies were excluded if: 1) there were insufficient data to extract the number of subjects in each group; 2) there were fewer than five subjects in either the patient group or the comparison group; 3) the comparison groups consisted of patients with minor non-psychiatric illnesses; 4) the patient group in the schizophrenia studies consisted of subjects with child-onset schizophrenia or chronic schizophrenia beyond the stage of first-episode psychosis; 5) the studies used region-of-interest volumetric analyses, or the deformation- or tensor-based volumetric methods for measuring regional brain volumes; and 6) the data contributed to another publication, in which case the publication with the largest group size under study was selected.

### Data abstraction

Data were extracted from two independent investigators (BO and IEW) and were double-checked. Information gathered from the studies included the authors, year of publication, demographic variables (number of subjects, age at baseline and gender), illness variables (diagnosis and duration of illness), and the reported stereotactic coordinates related to the comparisons between structural MRI scans of patients and controls from every selected study. Co-ordinates that were reported in the stereotactic space of the Montreal Neurological Institute (MNI) were converted to Talairach coordinates using the Lancaster transform (icbm2tal) in the GingerALE 2.0 program [29]. Talairach co-ordinates that had been generated by the Brett transform applied to statistical parametric mapping MNI co-ordinates were transformed back to MNI space in GingerALE and then to Talairach space using the Lancaster transform.

### Statistical analysis

Meta-analyses were performed using the Talairach stereotactic coordinates derived from the included studies. Meta-analyses were carried out using GingerALE 2.0 [29]. This uses the technique of Activation Likelihood Estimation (ALE) [25] and permits weighting of studies in the meta-analysis, e.g. based on sample size. This modification of ALE treats the spatial conjunction of co-ordinates from separate studies as more significant than conjunction of co-ordinates from a single study. The probability values can then be interpreted on an image-wide basis after correction for multiple testing using the False Discovery Rate, a method which controls the proportion of type 1 errors (false positives) among significant results [30].

Descriptive meta-analyses were then performed to identify the distribution of brain changes in FES and FTLD, when compared to control subjects. The overlap of these two distributions was measured.

### Spatial overlap testing

We tested the overlap of the FTLD co-ordinates with the FES spatial map generated by ALE using a randomisation method based on our previous work hybridising ALE spatial techniques with GSMA (Genome Scan Meta-Analysis) statistical methods [31].

For each FTLD study, the reported loci of maximal anatomical difference were modeled as the peaks of three-dimensional Gaussian probability density functions with full-width half-maximum of 7 mm, within a brain mask of size N of linear dimension 2 mm. The voxels in this probability image were then ranked from N (highest probability) to 1 (lowest probability), giving voxels of equal probability a mean rank. This created a rank image for each study which was smoothed with a 7 mm Gaussian filter. This image was masked with the First Episode Schizophrenia spatial map generated by ALE and the total value of ranks within the mask was calculated. The total for all studies of ranks within the mask was calculated (‘FES Mask Total’).

A null distribution for the FES Mask Total result was derived by 1000 permutations of the same process, but using an equal number of co-ordinates for each study derived from a random uniform distribution of coordinates within the brain mask. The probability of a FES Mask Total under the null hypothesis was calculated as the proportion of permutations giving a value equal or greater than the actual value.

The data set being tested was included in the ranking of all known outcomes. The significance threshold was set at p < 0.05.

## Results

**Figure 1:**
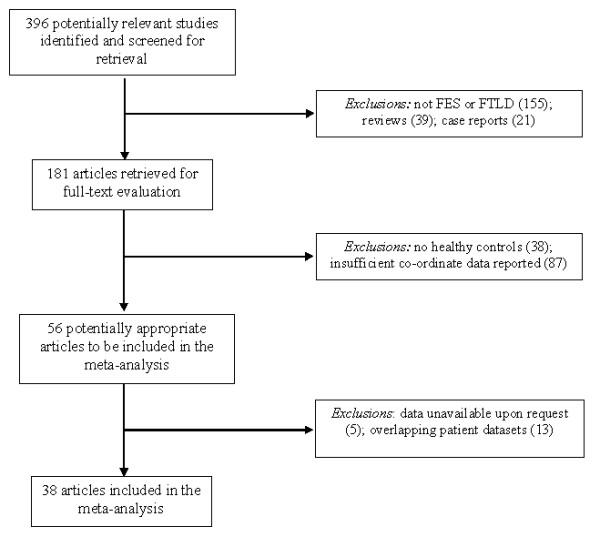
Study flow and reasons for exclusion.

The electronic literature search of the three databases yielded 396 articles, of which 181 were retrieved in full-text format. 56 studies were identified as being potentially appropriate to be included in the meta-analysis, and the inclusion and exclusion criteria was used throughout the selection process to yield 38 articles appropriate for use in the meta-analysis. Figure 1 displays a flow diagram that shows the reasons for exclusion at each stage of the selection process.

A total of eighteen FES studies and twenty FTLD studies were identified for inclusion in the meta-analysis (Table 1), having published MRI co-ordinates of gray matter changes in FES or FTLD patients and healthy controls [32-69]. The FES studies included a total of 555 patients and 621 comparison subjects, and the FTLD studies included a total of 311 patients and 431 comparison subjects. Among the FES patient group, the smallest size sample was 13 schizophrenia patients [44], and the largest size sample was 68 schizophrenia patients [40]. Among the FTLD patient group, the smallest size sample was 6 patients [50,62], and the largest size sample was 51 patients [60]. All included studies were published from 2000 to 2010.

**Table 1:** Studies included in the Meta-analyses

**First author, Year of publication**	**Disorder type**	**Number of patients**	**Number of controls**	**Mean age of patients (yrs)**	**Mean age of controls (yrs)**
**FIRST EPISODE SCHIZOPHRENIA**					
Berge, 2010 [32]	PNOS (FE)	21	20	24.8	25.3
Chua, 2007 [33]	SZ, SZF, BPSY (FE)	26	38	32.0	33.0
Douaud, 2007 [34]	SZ (FE)	25	25	16.5	16.2
Ebdrup, 2010 [35]	SZ (FE)	38	43	26.2	26.9
Jayakumar, 2005 [36]	SZ (FE)	18	18	24.9	25.7
Job, 2002 [37]	SZ (FE)	34	36	21.4	21.2
Kasparak, 2007 [38]	SZ (FE)	22	18	23.7	24.1
Kubicki, 2002 [39]	SZ (FE)	16	18	26.0	24.0
Lui, 2009 [40]	SZ (FE)	68	68	24.2	24.7
Meda, 2008 [41]	WPIC	22	21	25.1	26.2
Molina, 2010 [42]	SZ (FE)	30	41	25.8	29.4
Price, 2010 [43]	SCZ, SZA (FE)	48	47	26.2	24.8
Salgado-Pineda, 2003 [44]	SZ (FE)	13	13	23.8	23.4
Schaufelberger, 2007 [45]	SZ, SZF (FE)	62	94	27.6	30.2
Venkatasubramanian, 2010 [46]	SZ (FE)	30	27	30.1	27.4
Whitford, 2005 [47]	SZ (FES)	41	47	19.8	19.3
Witthaus, 2009 [48]	SZ (FES)	23	29	26.4	25.7
Yoshihara, 2008 [49]	SZ (FES)	18	18	15.8	15.8
**Total (mean)**		**555 (30.8)**	**621 (34.5)**	**(24.4)**	**(24.5)**
**FRONTO-TEMPORAL LOBAR DEGENERATION**					
Adlam, 2006 [50]	FTLD: SD, fPPA	6	12	62.8	65.0
Boccardi, 2005 [51]	FTLD	9	26	62.0	69.0
Boxer, 2003 [52]	FTLD: SD	11	15	66.2	69.6
Chang, 2005 [53]	FTLD: FTLD&ALS vs. ALS	10	10	64.5	49.9
Desgranges, 2007 [54]	FTLD: SD	10	17	65.7	65.8
Gee, 2003 [55]	FTLD	29	12	65.1	68.5
Gorno-Tempini, 2004 [56]	FTLD: SD	10	10	63.0	69.1
Grossman, 2004 [57]	FTLD	29	12	65.1	68.5
Kanda, 2008 [58]	FTLD: bvFTD	13	20	64.9	65.2
Kim, 2007 [59]	FTLD	14	61	63.3	68.0
Libon 2009 [60]	FTLD: bvFTD	51	43	61.3	n/av: age-matched
Massimo 2009 [61]	FTLD: disinhibition-predominant	4	10	63.6	64.1
Mummery 2000 [62]	FTLD: SD	6	14	60.5	62.0
Nestor 2003 [63]	FTLD: PNFA	7	10	71.5	65.9
Pardini 2009 [64]	FTLD: bvFTD	22	12	60.3	n/av: age-matched
Pereira 2009 [65]	FTLD: ubiquitin positive	9	25	64.0	63.8
Rabinovici 2007 [66]	FTLD	18	40	62.5	63.5
Rosen 2002 [67]	FTLD: bvFTD	20	20	61.8	62.3
Seeley 2008 [68]	FTLD: bvFTD CDR2-3	15	45	62.3	68.3
Williams 2005 [69]	FTLD	18	17	60.8	61.7
**Total (mean)**		**311 (15.6)**	**431 (21.6)**	**(63.6)**	**(65.0)**

All studies using voxel-based morphometry of gray matter compared a patient group with a comparison group. Abbreviations: *BPSY*, Brief Psychotic Disorder; *FE*, First episode; *PNOS*, Psychosis not otherwise specified; *SZ*, Schizophrenia; *SZA*, Schizo-affective disorder; *SZF*, Schizophreniform disorder; *WPIC*, Patients from the Western Psychiatric Institute and Clinic at the University of Pittsburgh; *FTLD*, Fronto-temporal Lobar Degeneration; *SD*, Semantic Dementia; *fPPA*, Fluent form of Primary Progressive Aphasia; *ALS*, Amyotrophic Lateral Sclerosis; *bvFTD*, behavioural / dysexecutive subtype of FTLD; *PFNA*, Progressive non-fluent Aphasia; *CDR*, Clinical Dementia Rating (range: 0.5 – 3); n/av, data not available.

### ALE meta-analyses

**Figure 2:**
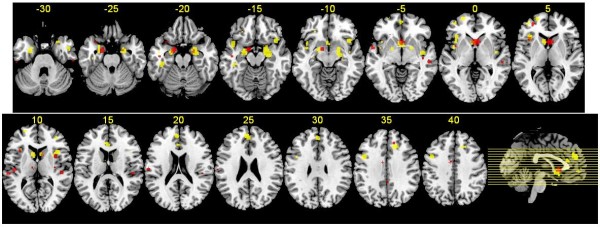
**Regions of gray matter change in FES and FTLD subjects.** Regions of gray matter decreases in FES subjects compared with controls (red), regions of gray matter decreases in FTLD subjects compared with controls (yellow), and overlapping regions of gray matter decreases controls (orange), displayed on a brain template. The left side of the image represents the left side of the brain. The Talairach level (z co-ordinate) is given above each horizontal slice.

Eighteen FES studies included 185 co-ordinates of gray matter decreases. Meta-analysis of this data identified gray matter decreases in regions including bilateral head of caudate nucleus, bilateral insula, bilateral amygdala/uncus region and bilateral superior temporal region (Figure 2).

Twenty FTLD studies provided 260 co-ordinates of gray matter decreases. Meta-analysis of this data identified gray matter decreases in regions including bilateral head of caudate nucleus, bilateral insula, bilateral amygdala/uncus region and bilateral superior temporal region (Table 2, Figure 2).

**Table 2:** Regions of gray matter decreases in FES and FTLD subjects compared with controls

**Cluster**	**Region**		**Talairach coordinates**			**Value**	**Volume (mm**^**3**^**)**
			**x**	**y**	**z**		
**FIRST EPISODE SCHIZOPHRENIA**							
1	R/L	Caudate	2	14	0	0.0046	1624
	R	Caudate	10	10	12	0.0017	
2	L	Uncus	−18	−2	−22	0.0031	1272
	L	Amygdala	−12	0	−14	0.0028	
3	R	Superior temporal gyrus	48	−24	10	0.0019	400
	R	Superior temporal gyrus	48	−26	16	0.0018	
4	L	Insula	−36	20	6	0.0025	376
5	L	Transverse temporal gyrus	−46	−18	10	0.0021	288
6	R	Middle temporal gyrus	54	−26	−2	0.0022	272
7	L	Superior temporal gyrus	−58	−26	12	0.0021	272
8	L	Superior temporal gyrus	−56	2	−4	0.0021	216
9	R	Insula	36	8	10	0.0021	216
10	R	Parietal lobe: Postcentral gyrus	54	−20	44	0.0022	216
11	L	Frontal lobe: Middle frontal gyrus	−30	50	4	0.002	200
12	L	Frontal lobe: Inferior frontal gyrus	−22	28	−6	0.0018	184
13	L	Parietal lobe: Postcentral gyrus	−60	−18	20	0.002	184
14	R	Amygdala / Uncus	20	−4	−22	0.002	168
15	L	Superior temporal gyrus	−32	14	−22	0.0019	152
16	L	Inferior temporal gyrus	−46	−14	−18	0.002	152
17	L	Cingulate gyrus	−8	−4	38	0.002	152
18	R	Insula / Claustrum	34	−14	12	0.0018	136
19	R	Medial frontal gyrus (BA 8)	14	34	36	0.0017	136
20	L	Cingulate gyrus	0	−42	38	0.0016	112
21	R	Medial frontal gyrus (BA 6)	6	6	48	0.0017	112
**FRONTO-TEMPORAL LOBE DEGENERATION**							
1	R	Amygdala / Uncus	22	−4	−20	0.0029	3744
	R	Globus Pallidus region	20	2	−4	0.0024	
	R	Hippocampus / Parahippocampus	28	−20	−10	0.0015	
2	L	Amygdala / Parahippocampus	−22	−6	−16	0.0027	2336
	L	Globus Pallidus region	−18	0	−6	0.0025	
	L	Uncus	−26	−4	−28	0.002	
3	L	Anterior cingulate	−2	10	−4	0.0024	2144
	L	Caudate	−8	10	10	0.0022	
	L	Caudate	−6	2	14	0.0015	
4	L	Frontal lobe: Medial frontal gyrus (BA 9)	−4	48	24	0.003	1256
5	L	Frontal lobe: Inferior frontal gyrus	−36	24	4	0.0022	936
	L	Insula	−38	14	0	0.0016	
6	L	Superior temporal gyrus	−42	12	−14	0.0021	896
	L	Superior temporal gyrus	−44	2	−12	0.0018	
7	R	Frontal lobe: Inferior frontal gyrus	42	16	−12	0.003	880
8	L	Frontal lobe: Middle frontal gyrus	−44	8	38	0.0032	784
9	R	Frontal lobe: Inferior frontal gyrus	42	16	12	0.0025	768
10	L	Frontal lobe: Superior frontal gyrus	−22	60	2	0.0023	704
	L	Frontal lobe: Superior frontal gyrus	−24	56	12	0.0017	
11	R	Cingulate gyrus	16	26	36	0.0025	576
12	R	Insula	42	−6	−4	0.0021	568
13	L	Temporal lobe: fusiform gyrus	−42	−30	−18	0.0027	520
14	L	Frontal lobe: Middle frontal gyrus	−22	18	50	0.0027	496
15	L	Anterior cingulate	−2	32	16	0.002	400
16	R	Caudate	8	12	12	0.0024	376
17	R	Middle temporal gyrus	48	2	−28	0.0024	312
18	L	Frontal lobe: Inferior frontal gyrus	−40	52	2	0.0024	232
19	L	Frontal lobe: Middle frontal gyrus	−28	34	−14	0.002	224
20	L	Insula	−42	2	0	0.0017	216
21	L	Parietal lobe (BA 7)	−20	−46	58	0.0023	216
22	L	Frontal lobe: Medial frontal gyrus (BA 10)	0	54	−4	0.0018	192
23	R	Superior temporal gyrus	28	14	−30	0.0016	176
24	L	Uncus	−34	−16	−34	0.0017	136
25	R	Cerebellum: Anterior lobe	48	−36	−28	0.0023	136
26	R	Frontal lobe: Middle frontal gyrus	36	44	8	0.0017	120
27	R	Uncus	28	−10	−32	0.0016	112

### Common changes in FES and FTLD

**Figure 3:**
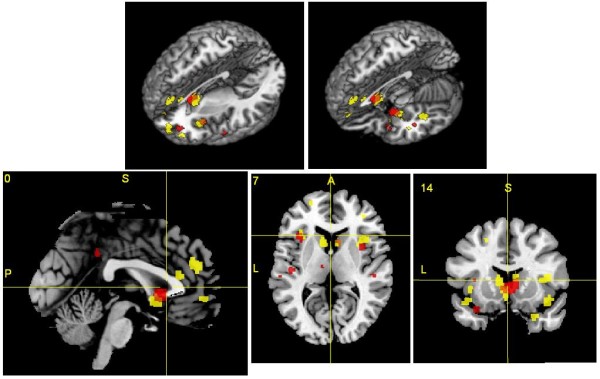
**Gray matter deficits in FES subjects (red) and FTLD subjects (yellow). Congruent changes in both disorders are shown in orange.** Brain changes are shown in three-dimensional views with brain sections removed (A, B), a sagittal plane (C), an axial plane (D) and a coronal plane (E). The Talairach co-ordinate for each plane is given above each image (C, D, E) (P = posterior, A = anterior, S = superior, L = left).

Gray matter decreases were present in both disorders in regions including bilateral caudate head, left insula and bilateral uncus region (Figure 3).

The spatial congruence between the disorders was estimated by percentage overlap. The FTLD map of gray matter decreases comprised 2450 voxels in a brain mask of 201069 voxels. The FES map of gray matter decreases comprised 888 voxels in a brain mask of 201069 voxels. The overlap between the FTLD and FES maps was 124 voxels. Therefore the overlap of FTLD within FES was 14% (compared to an expected overlap of 1.2% if the FTLD map was randomly distributed in the brain mask). The overlap of FES within FTLD was 5% (compared to an expected overlap of 0.4% if the FES mask was randomly distributed in the brain mask). Applying the spatial overlap testing method, the overlap of the FTLD co-ordinates with the FES spatial map generated by ALE was significant (p = 0.001).

## Discussion

**Figure 4:**
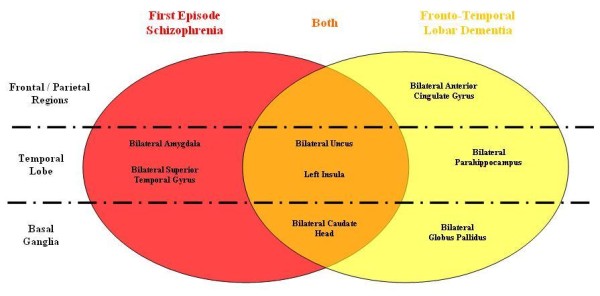
Venn diagram to summarise the distinct and overlapping regions of gray matter deficits found in FES and FTLD.

In this study, we identified overlap in the distribution of brain changes in First Episode Schizophrenia (FES) and Fronto-Temporal Lobar Degeneration (FTLD). We found that the two disorders involved gray matter deficits in common regions (p = 0.001). These included the basal ganglia (bilateral caudate head), paralimbic (left insula) and limbic (bilateral uncus) regions, as shown in Figure 4. To our knowledge, this is the first meta-analysis that compares neuro-structural profiles between FES and FTLD. Our study presents a novel method using ALE analyses to derive a statistical test for the chance overlap of the spatial distribution of the two disorders.

One approach to investigating the spatial distribution of brain changes in schizophrenia involves conceptualising the disorder as affecting several neural sub-systems which are individually affected in other neuro-psychiatric disorders with genetic or syndromal links to schizophrenia. For example, there is evidence that bipolar disorder and schizophrenia share susceptibility genes, and gray matter deficits of bipolar disorder overlap with those of schizophrenia but are more limited to paralimbic regions involved in emotion regulation [70-72]. Temporal lobe epilepsy is also associated with psychotic episodes with similarities to schizophrenia and is associated with gray matter deficits in limbic regions, overlapping with those of schizophrenia [73-75]. Autistic spectrum disorders and schizophrenia share gray matter deficits in the limbo-striato-thalamic circuitry [76], possibly reflecting shared genetic [77,78] and environmental [79-82] risk factors.

### Previous results of meta-analyses and structural neuro-imaging studies of FES and FTLD

These results confirm those of previous meta-analyses of the two disorders, using smaller samples of primary studies. Several previous meta-analyses using ‘region-of-interest’ analyses have examined gray matter deficits in schizophrenia [83,84], with consistent findings of medial temporal lobe atrophy [85-88]. Patients with FES also have smaller whole-brain volume, with greater lateral ventricular volume [89]. Frontal [90,91] and temporal [37] volumes have been reported to be smaller at first episode, and basal ganglia are also affected early in the illness [33,92]. The ALE method has also been used to investigate brain structural abnormalities in schizophrenia derived from studies using voxel-based morpometry analyses [1,93]. In a recent meta-analysis by Chan *et al.,* patients with FES had lower gray matter volumes in frontal, temporal, striatal, and cerebellar regions compared with both control subjects and people who are at genetically high-risk of developing schizophrenia [94]. They mapped progressive changes from the high-risk stage to the first-episode stage, clarifying potential markers for disease risk (anterior cingulate and right insula volume reduction) and for disease onset (caudate volume reduction) [94].

In FTLD, a meta-analysis of voxel-based morphometry studies found predominant frontal and temporal lobe involvement, with specific patterns of atrophy in the three clinical subtypes of FTLD, namely, FTD (frontotemporal dementia), SD (semantic dementia) and PNFA (progressive non-fluent aphasia) [95]. Correspondingly, diagnostic criteria for FTLD create a ‘triple dissociation’ of these subtypes with a high diagnostic accuracy clinically [96]. The clinical characteristics of each subtype of FTLD correspond well with the neuroanatomical deficits found.

### Functional significance of implicated regions and symptoms of FES and FTLD

The involvement of the basal ganglia (bilateral gray matter decreases in the caudate head in both disorders) agrees with Middleton and Strick’s prediction that abnormalities within cortico-striatal circuits may underlie neuropsychiatric symptoms [97,98]. Anatomical studies have revealed discrete connections between the basal ganglia and the cerebral cortex, reciprocally interconnecting a large and diverse set of cortical areas [99]. Dysregulation and abnormal dopaminergic transmission in these loops may contribute to hyperkinetic movements as well as cognitive impairments [100-102]. Robbins [103] proposed that the heterogeneous range of core symptoms associated with psychosis, appearing to be associated with a range of structural and functional abnormalities, might be explained by a frontal-striatal hypothesis, where an altered balance in the flow of information between through the basal ganglia could explain the seemingly disparate symptoms and characteristics of schizophrenia and psychotic episodes in FTLD. Our results indicate that the basal ganglia loop in particular incorporating the head of the caudate nucleus, are affected by FES and FTLD, which may at least partly explain their shared symptoms.

Our findings reveal that both disorders are characterised by reduced volumes of various paralimbic and limbic structures. Neuroanatomists and cytoarchitectologists have grouped the regions shown to be affected in FES and FTLD, namely the superior temporal gyrus (temporal pole), rostral and caudal anterior cingulate, posterior cingulate, orbital frontal cortex, insula, and parahippocampal regions, into the paralimbic cortex [104,105]. There is documented evidence to support paralimbic dysfunction in various psychiatric disorders, namely psychopathy [106], bipolar disorder [72], psychotic symptoms in depressive disorder [107] and attention deficit hyperactivity disorder [108]. Our results also indicate that both disorders are characterised by reduced caudate size, lesions of which cause impairments in learning, memory [109] and behaviour through the selection of appropriate sub-goals based on an evaluation of action-outcomes [110]. This may reflect the common symptomatology between the two disorders.

### Explanation for congruence

Various explanations need to be considered for the overlap in gray matter deficits between the two disorders. Firstly, the result could be due to coincidence. However, we have applied a statistical test to the spatial congruence which suggests that this is highly unlikely (the p value for the null hypothesis of a random distribution of FTLD co-ordinates within the FES spatial map was p = 0.001). Secondly, the symptoms of neuropsychiatric disorders are related to the anatomy of the brain pathology. Therefore, the selection of schizophrenia and FTLD, which share certain symptoms (as described above) may constrain the anatomical findings towards certain brain regions, in the absence of any other more meaningful connection between the neuropathology of the two disorders. The evidence that schizophrenia and FTLD co-occur in some families [6] suggests the possibility of a more fundamental connection between the two disorders. Thirdly, the neuropathology of the two disorders may be (at the neurochemical pathway level) distinct but the common network identified in this study may be ‘a final common pathway’ in the pathological process of both disorders, or a common reaction to such processes.

### Limitations

There are limitations of this meta-analysis. Firstly, by meta-analysing co-ordinates of maximum change from primary studies there is a loss of spatial information. This reduces the spatial resolution of the results. Secondly, as more primary studies are published the distribution of changes in each disorder may become more extensive as there is improved power to detect changes. Thirdly, there are alternative approaches for investigating spatial overlap of two disorders. For example, Yu *et al.,* used a post-hoc meta-analytic estimation of the extent to which gray matter compares with controls in bipolar disorder and schizophrenia by using a modification of the ALE method [71]. Lastly, the mean age of patients with FES was 24.4 years, whereas in FTLD, the mean age was 63.6 years (Table 1). There are no validated methods to account for these differences in an ALE meta-analysis, and it is possible that some of the results may have been affected by “normal” structural brain aging processes [111]. Anatomical likelihood estimation analyses are a relatively novel and changing technique, and as time progresses, standardisation of meta-analysis techniques will help researchers evaluate results from different studies more objectively.

### Future directions

In the future, localized gray matter deficits detected via the above analyses may be combined with those identified in activation studies of cognitive deficits in schizophrenia and FTLD, in order to understand the correlation between functional and structural connectivity in both disorders. For example, the structural MRI gray matter deficits could be used as nodes for a network analysis [112], as can now be done on individual scans [113], which may be utilized to investigate and compare functional connectivity changes in FES and FTLD.

## Conclusions

In summary, we reviewed data from 18 FES studies and 20 studies of FTLD that used voxel-based morphometry to identify common structural brain abnormalities. The brain regions found to be significantly affected included gray matter deficits in the bilateral caudate, left insula and bilateral uncus regions. The overlap in distribution of the disorders does not necessarily indicate a fundamental sharing of neurochemical pathology between FES and FTLD. However, we propose that the emerging genetic, pathological and clinical typology of FTLD may provide a model for the deconstruction of subtypes of schizophrenia.

## Competing interests

BO, IEW and SML disclose that they have no competing interests. EB is employed half-time by GlaxoSmithKline (GSK) and half-time by the University of Cambridge and is a stockholder in GSK.

## Authors’ contributions

BO and IEW devised the study and wrote the first draft of the manuscript. BO ascertained the studies and extracted the data. IEW checked and analysed the data. EB and SML participated in the design and coordination and helped to draft the manuscript. All authors read and approved the final manuscript.

## Pre-publication history

The pre-publication history for this paper can be accessed here:

http://www.biomedcentral.com/1471-244X/12/104/prepub
